# Elliptical shadow on the cervical spinal cord

**DOI:** 10.1002/jgf2.417

**Published:** 2021-01-25

**Authors:** Nozomi Nishikura, Ryuichi Ohta, Chiaki Sano

**Affiliations:** ^1^ Community Care Unnan City Hospital Unnan Japan; ^2^ Department of Community Medicine Management Faculty of Medicine Shimane University Izumo Japan

**Keywords:** chiari type 1 malformation, elderly, rural hospital

## Abstract

Chiari type 1 deformation can show progressive degenerative neurological presentation in the elderly.

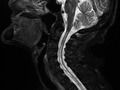

An 87‐year‐old woman with full activity of daily life (ADL) was admitted to a community hospital with the chief complaint of right lower leg pain. Redness and swelling were observed in her right knee. Following joint aspiration, the patient's initial diagnosis was pseudogout, which was treated using nonsteroidal anti‐inflammatory drugs and colchicine, resulting in improvement in pain. Thereafter, the patient underwent rehabilitation prior to discharge from the hospital. However, the muscles of the extremities gradually became atrophic in 2 months, which inhibited improvement in the patient's ADL. Cervical magnetic resonance imaging (MRI) performed to investigate the symptoms clarified the pathological findings; syringomyelia was noted with mild change in osteoarthritis in the T2 enhanced image (Figure 1). Widening of the cervical intraspinal space with a horizontally elliptical shape was observed in the T2 enhanced image (Figure 2). The patient was referred to a neurosurgeon and was diagnosed with Chiari type 1 malformation coexisting with osteoarthritis. Chiari malformation was first described by John Cleland in 1883 and later classified into four groups by Hans Chiari in 1991.^1^ Chiari type 1 malformation is mainly characterized by herniation of the cerebellar tonsils into the foramen magnum.^1^ It is often associated with syringomyelia and presents with features of spinal cord lesions. In Japan, the prevalence of syringomyelia is 1.94 per 100 000.^2^ Clinically, Chiari malformation type 1 can be asymptomatic until twenty or 3 years old. It is more frequently caused by acquired factors, such as adhesive arachnoiditis in late adulthood. However, in this case, no vertebral abnormalities were found, which is considered rare, as only a few cases have ever been reported.^3^, ^4^ The patient's symptoms gradually progressed, and the right knee joint and bilateral ankle joints became spastic. The patient declined treatment for spasticity, and she was transferred to a nursing home for palliative care. During follow‐up, her bilateral legs became spastic, and the physical examination showed bilateral positive Babinski reflex. Regarding the patient's ADL, she became completely bedridden. The progression of the pyramidal symptoms of extremities without vertebral deformities can facilitate the diagnosis of this disease.^3^ Though Chiari malformation type I is rare among older patients, the gradual progression because of aging can be possible.^4^ Notwithstanding no definitive treatment among the elderly, a precise diagnosis before the progression to the state of completed bedridden through the effective collaboration between general physicians and other specialists can help patients and their families to accept the symptoms and discuss advance care planning, alongside the methods for coping with the symptoms.^5^ A clear diagnosis in cases of rare diseases should be highlighted as one of the competencies required in general and family physicians for a better quality of life and longer life span of patients.

**FIGURE 1:**
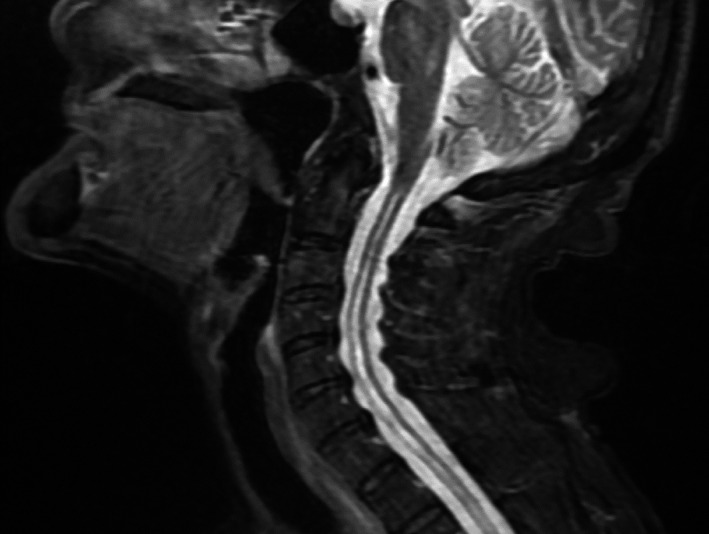
T2 enhanced sagittal image of the neck. Syringomyelia spreads in the cervical spinal cord

**FIGURE 2:**
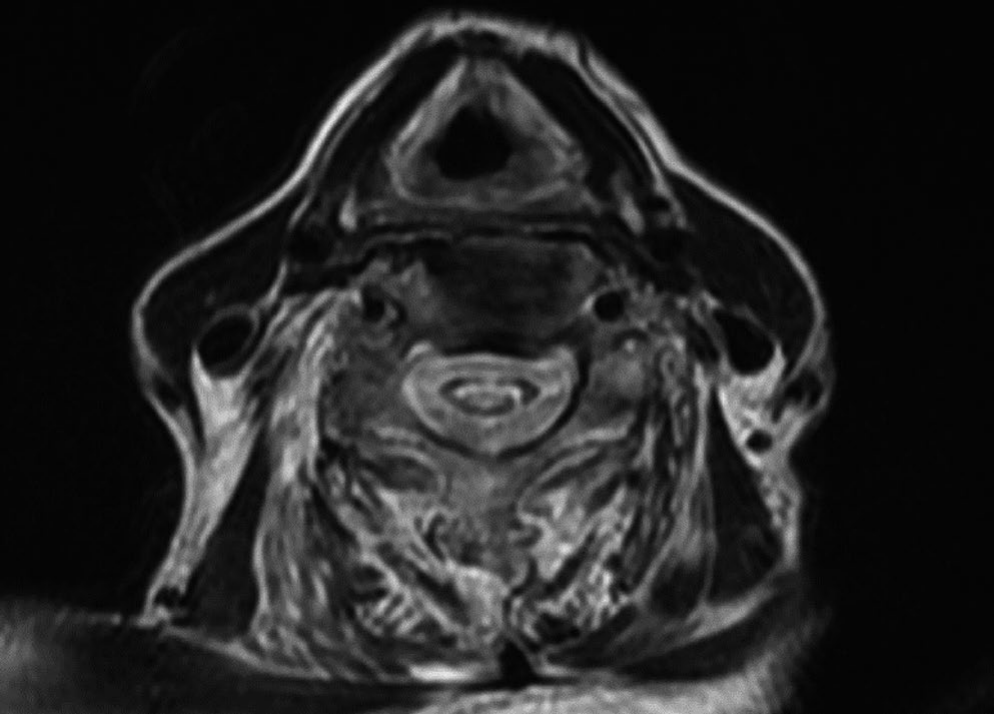
T2 enhanced axial image of the neck. Syringomyelia is observed as elliptical in the axial image

## CONFLICT OF INTEREST

The other authors have stated explicitly that there are no conflicts of interest in connection with this article.

## PATIENT CONSENT

Patient consent was obtained before publishing this case report.
